# Growth Hormone Influence on the Morphology and Size of the Mouse Meibomian Gland

**DOI:** 10.1155/2016/5728071

**Published:** 2016-02-14

**Authors:** Yang Liu, Erich Knop, Nadja Knop, David A. Sullivan, Edward O. List, John J. Kopchick, Wendy R. Kam, Juan Ding

**Affiliations:** ^1^Schepens Eye Research Institute, Massachusetts Eye and Ear, Harvard Medical School, Boston, MA 02114, USA; ^2^Ocular Surface Center, Charité-Universitätsmedizin Berlin, 10117 Berlin, Germany; ^3^Edison Biotechnology Institute, Ohio University, Athens, OH 45701, USA

## Abstract

*Purpose*. We hypothesize that growth hormone (GH) plays a significant role in the regulation of the meibomian gland. To test our hypothesis, we examined the influence of GH on mouse meibomian gland structure.* Methods*. We studied four groups of mice, including (1) bovine (b) GH transgenic mice with excess GH; (2) GH receptor (R) antagonist (A) transgenic mice with decreased GH; (3) GHR knockout (−/−) mice with no GH activity; and (4) wild type (WT) control mice. After mouse sacrifice, eyelids were processed for morphological and image analyses.* Results*. Our results show striking structural changes in the GH-deficient animals. Many of the GHR−/− and GHA meibomian glands featured hyperkeratinized and thickened ducts, acini inserting into duct walls, and poorly differentiated acini. In contrast, the morphology of WT and bGH meibomian glands appeared similar. The sizes of meibomian glands of bGH mice were significantly larger and those of GHA and GHR−/− mice were significantly smaller than glands of WT mice.* Conclusions*. Our findings support our hypothesis that the GH/IGF-1 axis plays a significant role in the control of the meibomian gland. In addition, our data show that GH modulates the morphology and size of this tissue.

## 1. Introduction

Meibomian glands play a critical role in the health and well-being of the eye. These tissues, which are relatively large sebaceous glands, secrete a lipid mixture (i.e., meibum) that promotes clear optical surface for the cornea, interferes with bacterial colonization, and retards tear overflow [1–4]. These secretions also enhance the stability and prevent the evaporation of the tear film [1, 3, 5]. Conversely, meibomian gland dysfunction (MGD) and the resulting meibum insufficiency destabilize the tear film, increase its evaporation and osmolarity, and are the most common cause of dry eye disease (DED) [1, 6–10], which afflicts 40 million Americans [11]. In fact, a recent study found that 86% of qualified DED patients show signs of MGD [12].

The pathophysiology of human MGD has been linked to several risk factors, especially aging [1, 13]. However, the molecular mechanisms that underlie the impact of these risk factors and the etiology of human MGD are largely unknown. This lack of information, in turn, has hampered the generation of safe and effective therapies for the treatment of MGD. There is no cure for MGD.

We hypothesize that identification of factors that control the physiology and pathophysiology of the meibomian gland will permit the development of new treatments for MGD. We further hypothesize that the growth hormone (GH)/insulin-like growth factor 1 (IGF-1) axis is such a factor. GH is a pituitary hormone that acts on multiple tissues to promote their function ultimately resulting in growth. Notably, GH induces the expression of IGF-1, which mediates many GH actions via endocrine, paracrine, and autocrine pathways [14]. Together, the GH/IGF-1 axis is a primary driving force of mammalian growth and a conserved regulator of aging in multiple species [15].

In support of our hypothesis, we have discovered that IGF-1 activates the PI3K/AKT and forkhead box O1 signaling pathways, stimulates the proliferation, increases the expression of sterol regulatory element-binding protein, and promotes lipid accumulation in human meibomian gland epithelial cells [16, 17]. Moreover, we have discovered that an antibody to the IGF-1 receptor, figitumumab, blocks the IGF-1-induced cellular signaling and lipid accumulation [16], which may account for this anticancer drug's induction of DED [18].

To continue to test our hypotheses, we sought in the present study to determine the effect of GH on mouse meibomian gland morphology and structure.

## 2. Materials and Methods

### 2.1. Mice

We utilized four groups of mice, including (a) male bovine (b) GH transgenic mice (bGH; 3.5 months of age) with excess GH and IGF-1 signaling, which are a model of acromegaly; (b) male GH receptor (R) gene knockout (−/−) mice (GHR−/−; 2.7 months of age) with no GH and low levels of IGF-1, which are a model of Laron Syndrome; (c) male and female GHR antagonist (A) transgenic mice (GHA; 5-6 months) with decreased GH and IGF-1 signaling, which are a model of GH deficiency; and (d) age- and sex-matched wild type (WT) littermate control mice in the same background strain (C57BL/6J) as the other three groups [19, 20]. The phenotypes of these mouse models have been extensively characterized, including their body size, body composition, glucose metabolism, and lifespan [21–28]. In brief, bGH mice are very large, lean, and insulin-resistant and have a shortened lifespan. In contrast, GHA and GHR−/− mice are dwarf and obese, and the GHR−/− mice are extremely insulin sensitive and live significantly longer than their littermate controls [21–28]. All three mouse lines used were developed at Ohio University and all animal procedures were approved by the Institutional Animal Care and Use Committee of Ohio University (Athens, OH). Following sacrifice by CO_2_ inhalation, mouse heads were removed, fixed in 10% neutral buffered formalin, stored at 4°C overnight, wrapped, and shipped on wet ice to the Schepens Eye Research Institute (Boston, MA). After arrival, upper and lower eyelids, attached to the globe, were removed as an entire unit and processed for morphological and image analyses.

### 2.2. Histology

Eyelids were dehydrated in a series of graded ethyl alcohols, then infiltrated, and embedded in glycol methacrylate resin (Technovit 7100, Electron Microscopy Sciences, Hatfield, PA, USA). Tissue samples were cut cross-sectionally in a Historange microtome (LKB Bromma, Germany) and sections (3 *μ*M) were placed onto glass slides. Each eyelid was cut at 5 to 8 different locations (3 to 5 sections/location), and each location was separated by at least 200 *μ*M. Sections were stained with Gill's #2 hematoxylin and eosin-y (Fisher Scientific, Pittsburgh, PA, USA) and overlaid with mounting media and glass coverslips.

### 2.3. Meibomian Gland Analyses

Tissue sections were evaluated with a Nikon eclipse E800 microscope (Micro Video Instruments, Inc., Avon, MA) at 100x and 400x magnification. The section in each tissue location containing the most clearly defined meibomian glands was imaged (*n* = 5 to 8 images/section/location). Visualized meibomian glands were outlined manually and enclosed areas were quantified with ImageJ (http://rsb.info.nih.gov/ij/). The area values were used to represent the size of the meibomian glands in upper and lower lids. One-tailed, unpaired* t*-test or two-way ANOVA statistics were performed with GraphPad Prism 5 (La Jolla, CA) to determine the significance (*p* < 0.05) of size differences between groups. The eyelid removal and histological examinations were conducted in a “blinded” manner, such that neither the sex nor the genotype of the sample was known by the investigator.

## 3. Results

**Figure 1:**
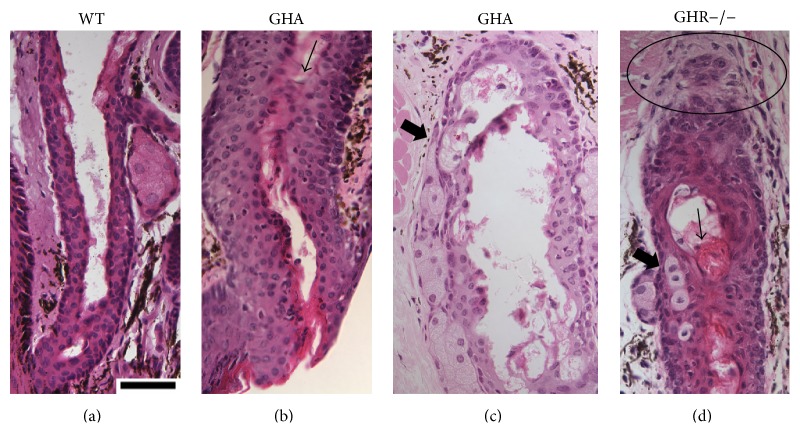
Meibomian gland morphology in male WT, GHA, and GHR−/− mice. (a) is from a WT mouse. (b) and (c) are from GHA mice. (d) is from GHR−/− mice. In (b) and (d), the arrows point to the cornified materials in the ducts. In (c) and (d), the thick arrows point to the secretory acini inserting into duct walls. In (d), the circle indicates possible gland dropout. All slides were stained with H & E, and the magnifications are 400x. The size bar in (a) is 50 *μ*m in length.

Our histological analyses demonstrate that the morphology of WT and bGH meibomian glands appear similar. In contrast, there are marked morphological changes in the GH-deficient animals. Many of the GHR−/− and GHA meibomian glands possessed hyperkeratinized and thickened ducts, ducts containing cornified materials, secretory acini inserting into duct walls, and poorly differentiated acini (Figure 1).

**Figure 2:**
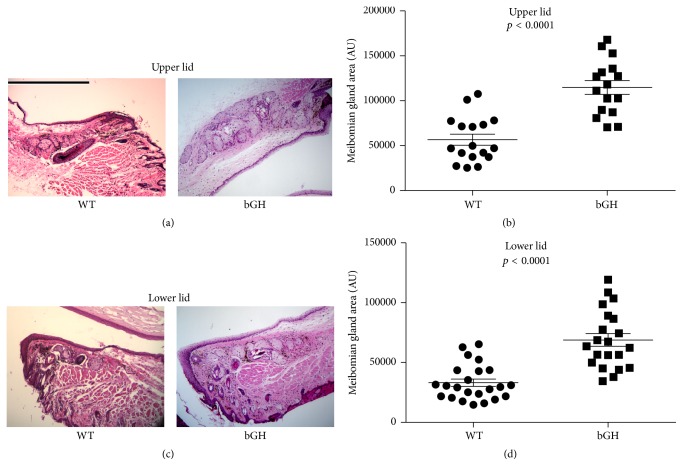
Meibomian gland morphology and size (i.e., area measurement) in male bGH and WT control mice. (a) and (c) H & E staining in the upper and lower lid tissue showing meibomian glands. (b) and (d) quantification of upper and lower lid meibomian gland size. Each data point represents the size of one meibomian gland, the horizontal bar represents the mean, and the error bar represents standard error of the mean (SEM). Data were pooled from *N* = 5 mice in each group. The magnifications for all photographs are 100x, and the size bar in (a) is 500 *μ*m in length.

**Figure 3:**
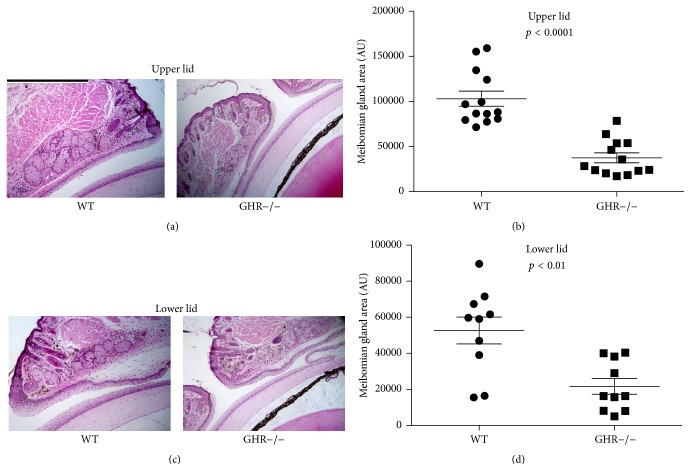
Meibomian gland morphology and size (i.e., area measurement) in male GHR−/− and WT control mice. (a) and (c) H & E staining in the upper and lower lid tissue showing meibomian glands. (b) and (d) quantification of upper and lower lid meibomian gland size. Each data point represents the size of one meibomian gland, the horizontal bar represents the mean, and the error bar represents SEM. Data were pooled from *N* = 3 mice in each group. The magnifications for all photographs are 100x, and the size bar in (a) is 500 *μ*m in length.

**Figure 4:**
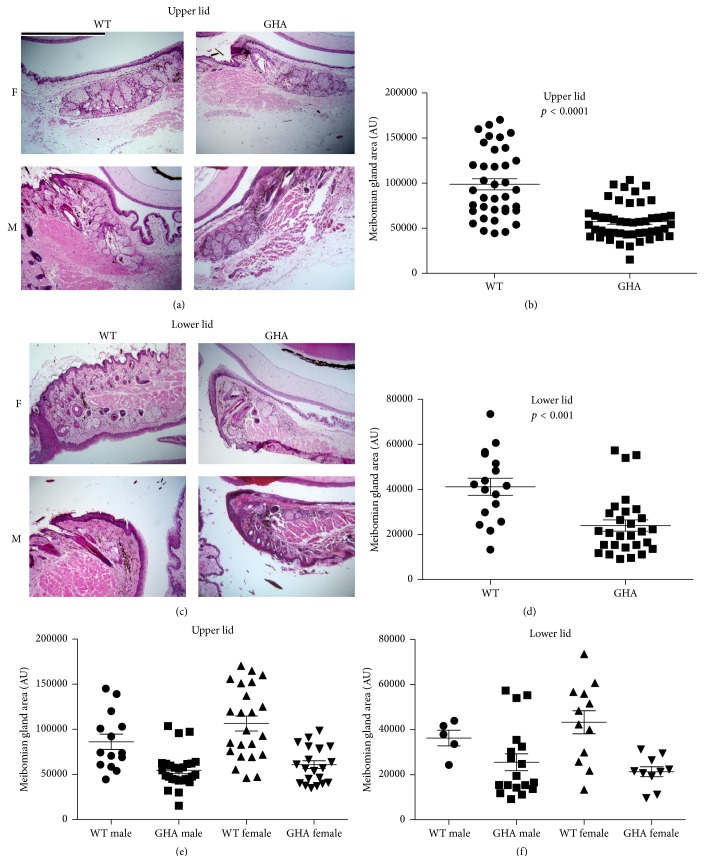
Meibomian gland morphology and size (i.e., area measurement) in both male and female GHA and WT control mice. (a) and (c) H & E staining in the upper and lower lid tissue showing meibomian glands. (b) and (d) quantification of upper and lower lid meibomian gland size without distinguishing sexes. (e) and (f) quantification of upper and lower lid meibomian gland size of both sexes. Each data point represents the size of one meibomian gland, the horizontal bar represents the mean, and the error bar represents SEM. Data were pooled from *N* = 5 mice in each group. The magnifications for all photographs are 100x, and the size bar in (a) is 500 *μ*m in length.

**Figure 5:**
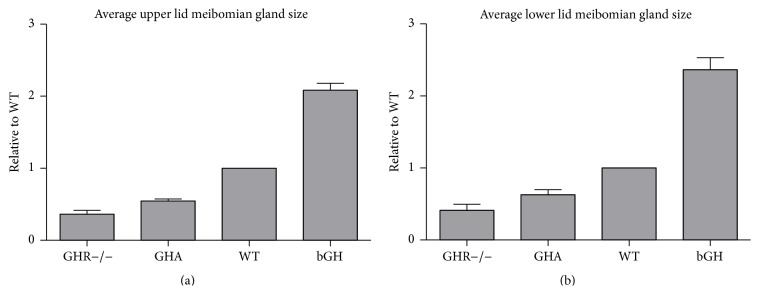
The ratio of average meibomian gland size relative to WT controls in GHR−/−, GHA, and bGH mice for both upper (a) and lower (b) eyelids. The WT in each group was set to 1. For the GHA group, the data for both sexes were pooled. Data shown are mean ratios ± SEM.

Image analyses showed that the sizes (i.e., area measurements) of bGH mouse meibomian glands were significantly larger than those of WT mice (Figure 2). The mean increase in size was more than 2-fold for both upper and lower lid meibomian glands (Figure 2). GHR−/− mice, on the other hand, had significantly smaller meibomian glands (Figure 3), with mean sizes 36% and 41% of the WT controls for upper and lower lids, respectively (Figure 5). The GHA mice also showed significantly smaller meibomian glands in both upper and lower lids (Figures 4(a), 4(b), 4(c), and 4(d)). The meibomian gland sizes of GHA mice relative to WT controls were both 58% for upper and lower lids (Figure 5). Interestingly, we also detected a significant size difference between male and female WT mice in the upper lid (*p* = 0.04), which was not present between male and female GHA mice (Figures 4(e) and 4(f)).

## 4. Discussion

Our findings demonstrate that decreased and/or disrupted GH action is associated with striking alterations in the morphology of mouse meibomian glands. The GHR−/− and GHA glands contained hyperkeratinized and thickened ducts, secretory acini inserting into duct walls, and poorly differentiated acini. The meibomian gland sizes (i.e., area measurements) of GHA and GHR−/− mice were also significantly smaller than those of age- and sex-matched WT controls. In contrast, meibomian gland sizes of bGH mice were significantly larger than those of WT mice, but the glandular morphology of bGH and littermate controls appeared similar. Our results support our hypothesis that the GH/IGF-1 axis plays a significant role in the control of the meibomian gland. More specifically, our data show that GH modulates the size of this tissue and that GH insufficiency leads to striking morphological changes.

Our discovery that GH regulates the meibomian gland was not unexpected. GH is known to play an important role in the growth of sebaceous glands elsewhere in the body [29]. Sebaceous glands express GH receptors and GH has been shown to directly induce the differentiation of sebaceous gland epithelial cells [30]. In addition, GH excess—as in acromegaly—has been linked to increased sebum production [31, 32], and GH insufficiency may lead to decreased sebaceous gland size and function [30, 33].

The morphological alterations associated with GH deficiency and resistance appear to be very clinically relevant for the eye. These changes, and in particular the hyperkeratinized ducts and meibocyte integration into the duct wall, are very similar to those observed in patients with obstructive MGD [1]. Indeed, ductal hyperkeratinization is a prominent characteristic of this disorder in humans [1]. Given that GH levels decline with aging, this decrease may contribute to the development of age-related MGD [1, 13].

The decreased size of meibomian glands in GHR−/− mice and GHA mice represents another characteristic of MGD, which often involves glandular atrophy and dropout [1]. Such a size decline is consistent with the finding of others [34–36], who reported reduced growth of orbital structures in decreased GH action (e.g., Laron Syndrome). In contrast, the larger size of meibomian glands in bGH mice is analogous to the observation that the acromegalic patients tend to have bigger sebaceous glands than normal people [37]. It will be of particular interest in the future to use volumetric techniques, such as meibography or three dimensional reconstruction [38], to determine whether the meibomian volumes in these mouse models reflect the area measurement differences found in this study.

Our observation that female WT mice have larger upper lid meibomian gland than the male WT mice is intriguing. We have previously found that sex differences exist in the morphology of the mouse meibomian gland and that these may be due to the influence of estrogen and progesterone [39]. It is possible that sexual dimorphism in glandular size may be attributed, at least in part, to the known ability of sex steroids to promote anabolic effects of GH [40, 41]. This might account for why sex-related differences were not observed in the meibomian glands of GHA mice. It is unclear why sex-associated variations were not discovered in the size of lower lid meibomian glands. There are many structural differences between the glands of the upper and lower lids [1], and these could theoretically extend to functional variations as well.

In conclusion, our results support our hypothesis that the GH/IGF-1 axis plays a significant role in the regulation of the meibomian gland. Our findings also suggest that the GHR−/− mice and GHA mice may serve as an MGD animal model, and the GH/IGF-1 axis may become a new target for the potential treatment of MGD.
